# Treatment of hypercholesterolemia: screening of *Solanum macrocarpon* Linn (Solanaceae) as a medicinal plant in Benin

**Published:** 2014

**Authors:** Tamègnon Victorien Dougnon, Honoré Sourou Bankolé, Jean Robert Klotoé, Maximin Sènou, Lauris Fah, Hornel Koudokpon, Casimir Akpovi, Tossou Jacques Dougnon, Phyllis Addo, Frédéric Loko, Michel Boko

**Affiliations:** 1 *Laboratory of Toxicology and Environmental Health, Interfaculty Center of Formation and Research in Environment for the Sustainable Development, University of Abomey-Calavi (UAC), 01 PO Box 1463 Cotonou, Benin*; 2 *Laboratory of Research in Applied Biology, Polytechnic School of Abomey-Calavi, University of Abomey-Calavi, 01 PO Box 2009 Cotonou, Benin*; 3 *Department of Animal Experimentation, Noguchi Memorial Institute for Medical Research (NMIMR), University of Ghana, PO Box LG 581 Legon, Ghana*

**Keywords:** *Cholesterolemia*, *Feeding*, *Regulation*, *Solanum macrocarpon*, *Wistar rats*

## Abstract

**Objective:** Hypercholesterolemia is the greatest risk factor for cardiovascular diseases. The present study is conducted to evaluate the lipid lowering activity of leaves and fruits of *Solanum macrocarpon, *a vegetable, on Wistar rats experimentally rendered hypercholesterolemic by Triton X-100.

**Materials and Methods:** The leaves and fruits were administered (p.o.) for 7 days to rats at doses of 400 and 800 mg/kg of body weight. Atorvastatin was used as reference treatment drug. The data were analyzed by the Brown-Forsythe ANOVA, Dunnett’s T3 multiple comparison test, and Dunnett’s t test. All tests were done at the 5% significance level.

**Results:** Administration of *S. macrocarpon* (fruits as well as leaves) resulted in a statistically significant decrease in total cholesterol, LDL-cholesterol, VLDL-cholesterol, and triglycerides in the treated groups compared with the untreated hypercholesterolemic group, regardless of the administrated doses. A significant increase in HDL-cholesterol was observed in the treated groups. Hepatic disorders due to the Triton have been corrected by *S. macrocarpon*.

**Conclusions**: This vegetable effectively suppresses experimental hypercholesterolemia in Wistar rats, suggesting a protective role in cardiovascular diseases. Its use by individuals at risk should be promoted.

## Introduction

Despite remarkable advances in medicine and research, there is an increase in cardiovascular diseases (Callias, 2007 ▶). It is one of the leading causes of death worldwide. They are responsible each year of 30% of mortality in the world. About ¾ occur in countries with low and middle incomes. Twenty-five million deaths are expected in 2020 (Rasheed and Hassan, 2007). Hypercholesterolemia is a metabolic condition that determines the onset of chronic degenerative diseases such as atherosclerosis (Devi and Sharma, 2004 ▶; Kim et al., 2008 ▶). It is characterized by the elevation of cholesterol and lipid parameters (LDL-cholesterol, triglycerides). It is estimated provoke about 4.4 million deaths (7.9%) in the world. Scientists often cite lifestyle: unhealthy diet, physical inactivity, lack of exercise, stress, smoking, and obesity as predisposing factors (Callias, 2007 ▶). 

There is hypercholesterolemia when cholesterol (LDL) in the blood is higher than the actual needs. The excess not used by the cells then tend to settle against the vessel walls, causing, if not recovered and returned to the liver by HDL, obstruction of the arteries, leading to infarction (Callias, 2007 ▶). Triglycerides are lipid molecules formed in the small intestine from fat consumed. They are also produced in the liver from excess sugar diet. Fats are not soluble in water; triglycerides need to combine with other substances -lipoproteins- to be transported throughout the body. 

There are three types of these triglyceride-containing lipoproteins: chylomicrons produced by the small intestine after meals, very low density lipoprotein, or VLDL (Very Low Density Lipoproteins) produced by the liver from sugars, and lipoproteins intermediate density or IDL (Intermediate Density Lipoproteins) from the conversion of VLDL. The gut releases chylomicrons and VLDL liver. These lipoproteins spread into the bloodstream where degradation system turns them into free fatty acids, which are in turn used by tissues as an energy source (Bruckert, 2013). The excess is stored as energy reserves in fat cells called adipocytes. The elevation of triglycerides promotes the formation of atheroma that increase cardiovascular and thrombotic risks (clotting), especially in the presence of other cardiovascular risk factors such as hypertension, physical inactivity, and obesity (Bruckert, 2013). Despite the difference in the distribution and lipoprotein metabolism between humans and laboratory rats, models of hypercholesterolemic Wistar rats are frequently used in lipid research (Kothiyal and Gupta, 2011 ▶). Many studies have been made to propose alternative treatment based on plant species (Kothiyal and Gupta, 2011 ▶; Reddy et al., 2011 ▶; Patel et al., 2012 ▶; Sodipo et al., 2012 ▶, Ghorbani, 2013 ▶). In Benin, a vegetable (*S. macrocarpon*), is reported to have cholesterol-lowering properties. 

The acute and medium toxicity assessment of *S. macrocarpon* revealed that it is safe (Dougnon et al., 2013a ▶-b ▶) with 3000 mg/kg at least. *S. macrocarpon* is traditionally used in Nigeria to treat hypercholesterolemic disorders so its purported efficacy as an anti-hypercholesterolemic was evaluated in this study. The effects of powdered leaves and fruits of *S. macrocarpon* on hypercholesterolemia induced in Wistar rats were assessed and then compared with those of a reference chemical product, Atorvastatin.

## Materials and Methods

Mature leaves and fruit of *S. macrocarpon* were used as the plant material in this study. They were purchased in July 2012 at Houéyiho located 6°21'20'' North Latitude and 2°21' 35'' East Longitude in Benin. It is the largest vegetable site of Benin (Chidikofan, 2010 ▶).

Albino Wistar rats were bred at the Laboratoire de Recherche en Biologie Appliquée (LARBA), University of Abomey-Calavi (UAC) at a constant temperature of 22±1° C with a 12-hour cycle of light and 12 hours of darkness. They were fed with pelleted feed and water *ad libitum*. Their weight was 160-200 grams and they were used to perform the *in vivo* tests.

The chemical Triton X-100 used to induce the disorder was obtained from Sigma Aldrich [CAS: 9002-93-1 (C_2_H_4_O)_n_C_14_H_22_O], while the drug, Atorvastatin, was purchased at a pharmacy [Manufactured by Ajanta Pharma (Mauritius) Limited, Digital Industrial Building, Goodlands, Mauritius]. Cyprex Diagnostics was the kit commercially available for measuring lipids.

**Powdered leaves and fruits of** ***S. macrocarpon***

Several samples of leaves and fruits were collected from twenty vegetable sites and pooled together in the laboratory. A specimen of the plant was identified and authenticated by the National Herbarium of Benin under No. AA 6423 / HNB July 31, 2012. The leaves were carefully washed with distilled water and then dried at ambient temperature of 16 °C for 17 days in LARBA while fruits were washed and finely cut into small pieces before being dried during 09 days. Leaves and dried fruits were ground for ten minutes using a commercial Moulinex blender. The obtained powders were sieved and stored in sterile containers. 

The leaves and fruits powders were dissolved in physiological saline for 24 hours and then stored until use (Giri et al., 2012 ▶).

**Evaluation of cholesterol-lowering properties of** ***S. macrocarpon***

The study was carried out in LARBA, University of Abomey-Calavi, Benin after an institutional approval. Before the implementation of the protocol, all rats were weighed. Six groups of five Wistar rats (1, 2, 3, 2 ', 3', and A) were treated with Triton X-100 (a substance used to induce hypercholesterolemia) at a single dose of 150 mg/kg body weight. The Triton X-100 was administered intraperitoneally and was chosen because of its convenience, availability, and especially the reproducibility of the animal model created (Kothiyal and Gupta, 2011 ▶). One additional group of five rats served as negative control (group B). They received no treatment with Triton X-100 but physiological water. Overall, there were seven groups of 5 rats. Seventy-two hours after induction of hypercholesterolemia with Triton X-100, the correction phase of its hypercholesterolemic effects was launched with the powdered fruits and leaves of *S. macrocarpon*. 

**Treatment of Wistar rats by fruits and leaves powders**

Group 1 received no corrective treatment (positive control) while groups 2 and 3 received 400 and 800 mg/kg body weight, respectively, of powdered fruits of *S. macrocarpon*. The 2' and 3', respectively, received 400 and 800 mg/kg body weight of powdered leaves of *S. macrocarpon*. Group A received a dose of standard drug (Atorvastatin 10 mg/kg body weight). The treatments were done orally every morning for 7 days. Twenty-four hours after the last day of treatment (i.e., the 8^th^ day), biochemical parameters (total cholesterol, triglycerides, HDL-C, LDL-C, and VLDL) were determined. In fact, by retro-orbital puncture, blood was collected in dry tubes. The determination of total cholesterol, HDL-C, and triglycerides was performed endpoint according to the methodology described by Deweerdt and Later (2009) ▶. LDL-C was calculated by the Friedewald formula (Djamel, 2007 ▶): LDL-C=Total Cholesterol-HDL-C-VLDL

VLDL cholesterol was estimated from the total serum triglycerides (Djamel, 2007 ▶): VLDL=Triglycerides/5

Two rats per group were dissected to assess liver histology according to the method used by Codo (2012) ▶. Histological tests were conducted at the Laboratoire d’Anatomie Pathologique et Cytopathologie (L.A.P.C).

**Statistical analysis**

The statistical package for the social sciences (SPSS), version 17, was used for data analysis. The data were initially examined for normality and homogeneity of variance by the explore procedure. Thereafter, the Brown-Forsythe ANOVA and Dunnett’s T3 multiple comparison test were used to test for differences in the groups on one hand and identify the group(s) eliciting the difference(s) on another hand, respectively. Subsequently, Dunnett’s t test was used to compare the controls with the treatment groups. All data were expressed as mean ± SD; p values < 0.05 were considered significant. GraphPad Prism 5 was used for the designing of the graphs.

## Results

Hypercholesterolemia was successfully established in all of the groups treated with Triton X-100. All of the hypercholesterolemic groups treated with Atorvastatin or *S. macrocarpon* showed a reduction in all hypercholesterolemia parameters (total cholesterol, LDL, VLDL, and triglyceride) and an increase in HDL. The mean differences were all statistically significant (p<0.05) with the exception of LDL (p=0.157).

**Figure 1:**
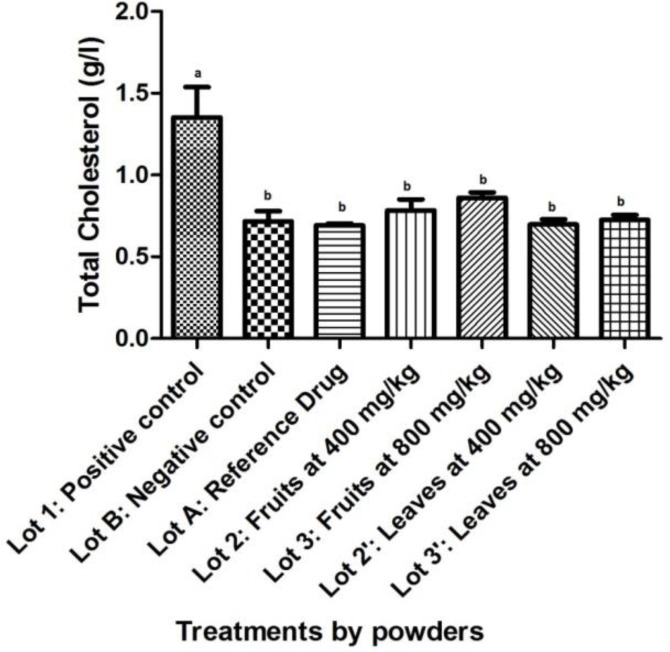
Effect of powdered *S. macrocarpon *on Total Cholesterol (averages with different letters are statistically different with a threshold of 5%).

With regard to total cholesterol, a significant decrease (p<0.05) was observed between the positive control and the other: the negative control, the batch treated with atorvastatin, and those treated with powders of fruits and leaves (Figure 1). Compared with the negative control, the average of total cholesterol from batches treated with powders of fruits and leaves showed no significant difference (p=0.981 for fruits at 400 mg/kg; p=0.655 for fruits at 800 mg/kg; p=1.000 for leaves at 400 mg/kg and p=1.000 for leaves at 800 mg/kg). No difference was noted when compared total cholesterol from batches treated with powders of fruits and leaves to Atorvastatin (p>0.05).

**0**

**Figure 2:**
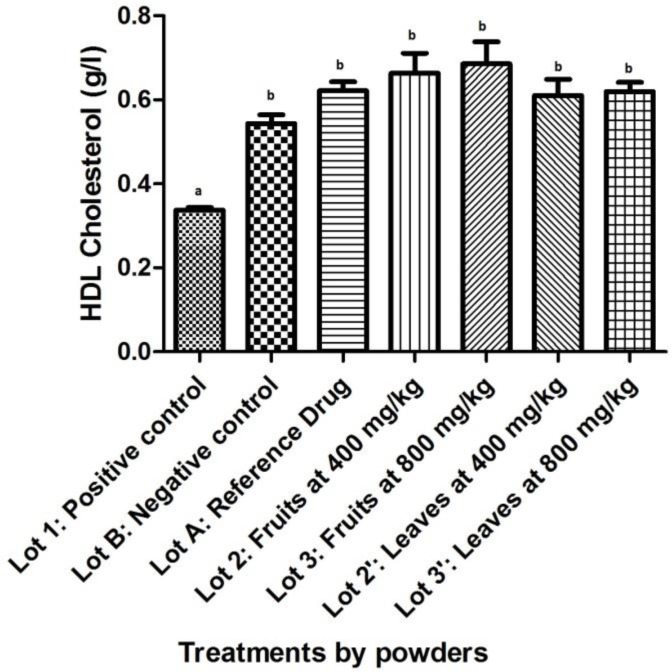
Effect of powdered *S. macrocarpon *on HDL-cholesterol (averages with different letters are statistically different with a threshold of 5%).

About HDL-cholesterol, a significant increase (p<0.05) was observed between the positive control and the other: the negative control, the batch treated with atorvastatin, and those treated with powders of fruits and leaves (Figure 2). Compared with the negative control, the average of HDL-cholesterol from batches treated with powders of fruits and leaves showed no significant difference (p=0.069 for fruits at 400 mg/kg; p=0.535 for leaves at 400 mg/kg, and p=0.397 for leaves at 800 mg/kg). At 800 mg/kg, the lot treated with powdered fruits showed a significant increase (p<0.05). No difference was noted when compared total cholesterol from batches treated with powders of fruits and leaves to Atorvastatin (p>0.05).

**Figure 3:**
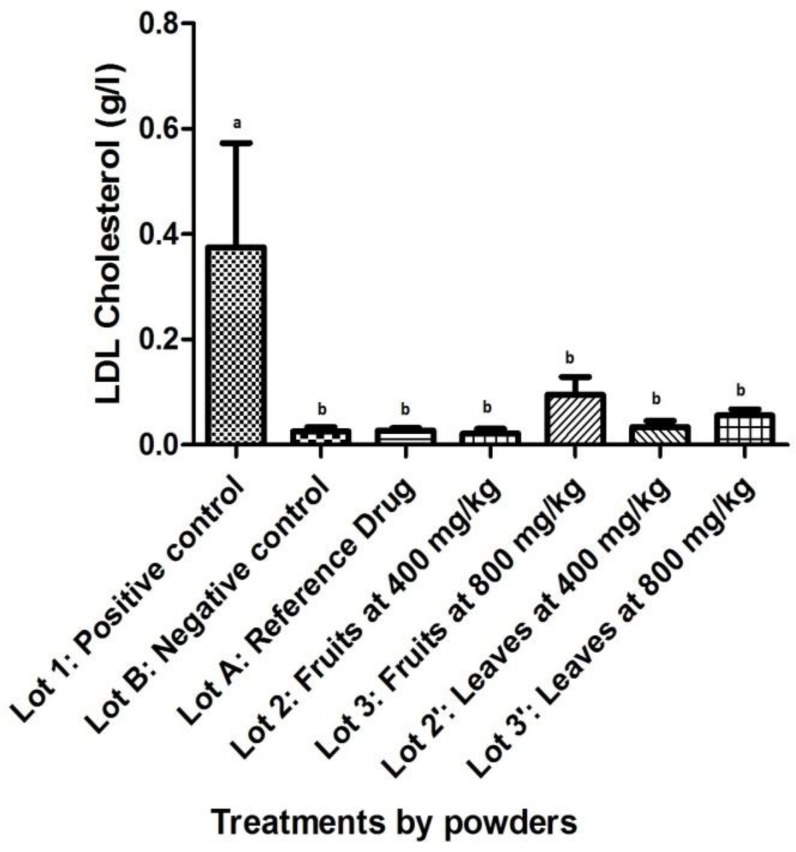
Effect of powdered *S. macrocarpon *on LDL-cholesterol (averages with different letters are statistically different with a threshold of 5%).

LDL-cholesterol peaked in positive control values compared with negative lot and treated groups [(p<0.05) (Figure 3)]. Compared with the negative control, LDL-cholesterol of batches treated with powders of fruits and leaves showed no significant difference (p=1.000 for fruits at 400 mg/kg; p=0.970 for fruits at 800 mg/kg; p=1.000 for leaves at 400 mg/kg, and p=1.000 for leaves at 800 mg/kg). The same remark was made with reference lot (p=1.000 for fruits at 400 mg/kg; p=0.972 for fruits at 800 mg/kg; p= 1.000 for leaves at 400 mg/kg, and p=1.000 for leaves at 800 mg/kg).

**Figure 4:**
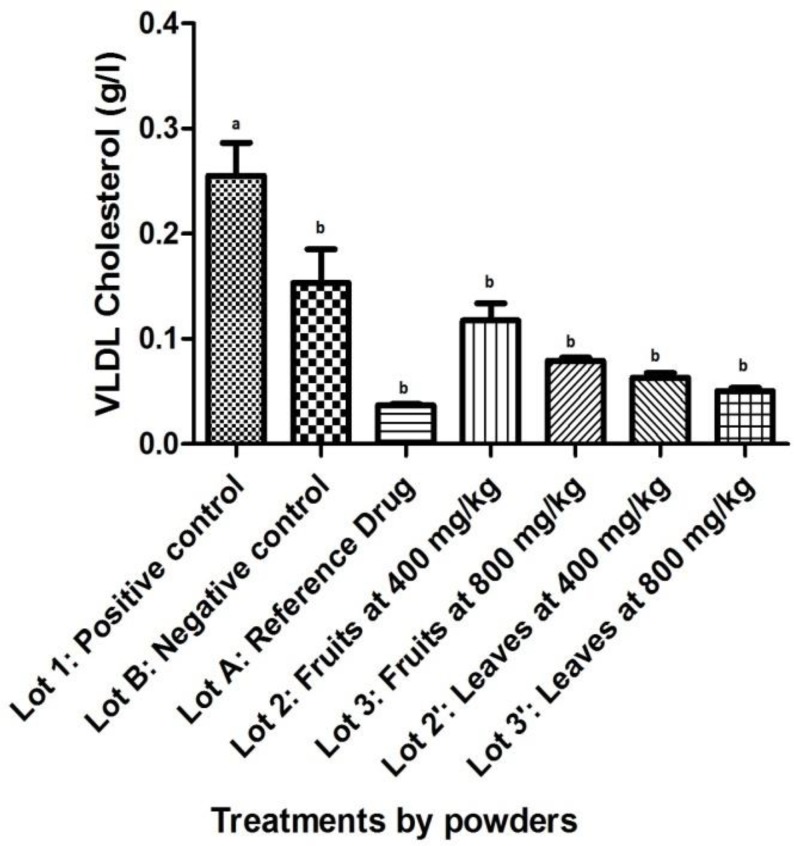
Effect of powdered *S. macrocarpon *on VLDL-Cholesterol (Averages with different letters are statistically different with a threshold of 5%).

About VLDL-cholesterol, there was a peak in the positive control’s group compared to other batches (p<0.05). A better decreasing was noted with to other groups when compared to negative control’s group (p<0.05). Although it was not significant, a decreasing was observed with the dose of 400 mg/kg (p=0.563 for powdered fruits). No significant difference was noted when compared reference’s group to those of powdered fruits and leaves (Figure 4).

**Figure 5:**
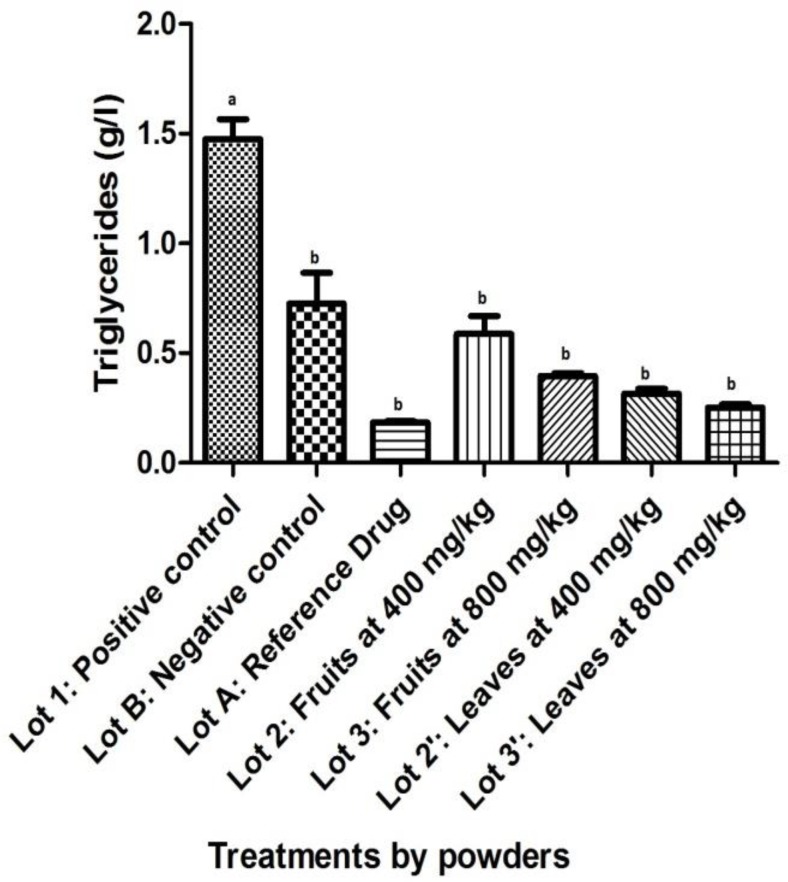
Effect of powdered *S. macrocarpon *on triglycerides (averages with different letters are statistically different with a threshold of 5%).

About triglycerides, a significant decrease (p<0.05) was observed between the positive control and the other: the negative control, the batch treated with atorvastatin and those treated with powders of fruits and leaves (Figure 5). Compared with the negative control, the average of triglycerides from batches treated with powders of fruits and leaves showed significant decreasing except for the group that received powdered fruits at 400 mg/kg (p=0.563). No difference was noted when compared triglycerides from batches treated with powders of fruits and leaves to Atorvastatin (p>0.05).

**Histological findings in the liver**

*Lot not intoxicated, Lot B (negative control)*

These animals had normal liver typical structure organized into lobules. Each lobule was centered on a central vein. The lobule was composed of hepatocytes spans more or less radial organized around the central vein. Between the bays were venous sinusoids. Space door with vessels and bile ducts were found at the crossroads of several lobules (Figure 6a).

**Animals intoxicated with Triton X-100, Lot 1 (positive control)**

The injection of Triton X-100 in Wistar rats resulted in a deterioration of the general architecture of the liver lobules, disruption of the sinusoids, and reduced sinusoidal lights. Some hepatocytes were dropsy and micro-vacuoles degeneration appeared in others (Figure 6b).

**Animals treated with 400 mg/kg of leaves (Lot 2 ')**

Treatment with 400 mg/kg of leaves restored the lobular structure. The sinusoids were back to normal and the radial arrangement of hepatic cords recovered. However, micro-vacuoles existed in several hepatocytes and indicated a persistent cellular damage (Figure 6c). 

**Animals treated with 800 mg/kg of leaves (Lot 3')**

Treatment with 800 mg/kg of leaves restored lobular architecture and venous sinusoids. There were persistant vacuoles in rare hepatocytes (Figure 6d).

**Figure 6a:**
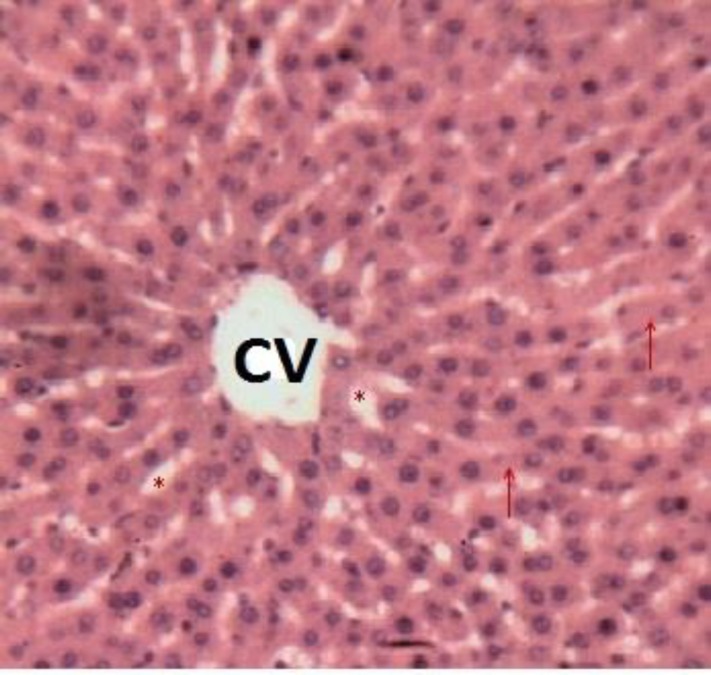
Histological appearance of the liver of rats in the negative control lot. Legend: Typical lobular structure. In the center is the Central Vein (CV). Asterisks (*) indicate the sinusoids venous and arrows denote the hepatocyte spans. Image taken at 10x magnification

**Figure 6b:**
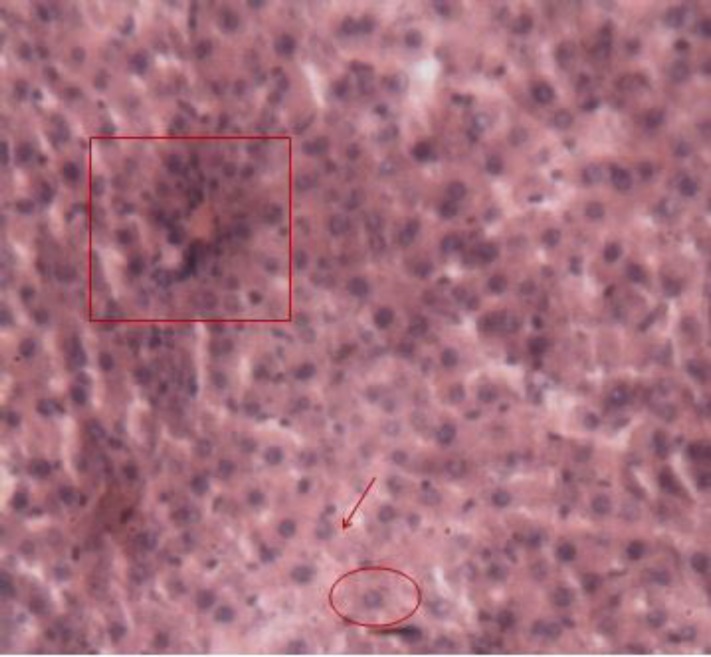
Histological appearance of the liver of rats in the positive control lot. Legend: Disorganized lobular architecture. An hydropic cell degeneration was surrounded and intracellular vacuole is indicated by an arrow. Space door was still recognizable (box). Image taken at 10x magnification

**Figure 6c:**
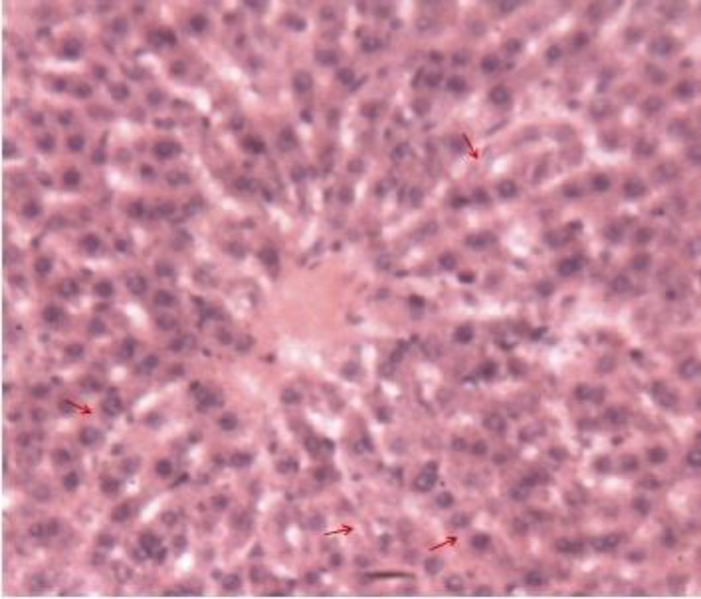
Histological appearance of the liver of rats treated with the leaves of *S. macrocarpon* (400 mg / kg). Legend: The arrows indicate the intracellular vacuoles. Image taken at 10x magnification

**Figure 6d:**
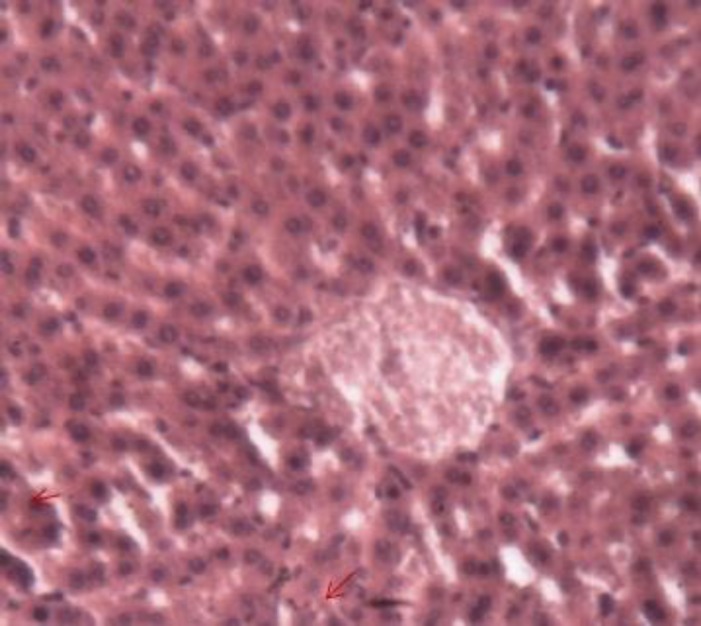
Histological liver of the rats treated with leaves at a dose of 800 mg/ kg. Legend: The arrows indicate the presence of vacuoles in rare hepatocytes. Image taken at 10x magnification

**Animals treated with fruit at 400 mg/kg (Lot 2), 800 mg/kg (Lot 3), and Atorvastatin (Lot A)**

In these different groups of rats, liver lobules were restored and resembled those of negative control’s lot. The vacuoles were very rare in hepatocytes and clearly visible sinusoidal lights were well placed. At the periphery of the lobules, spaces clearly identifiable doors were typical structure (Figures 6e, 6f, 6g, and 6h).

**Figure 6e:**
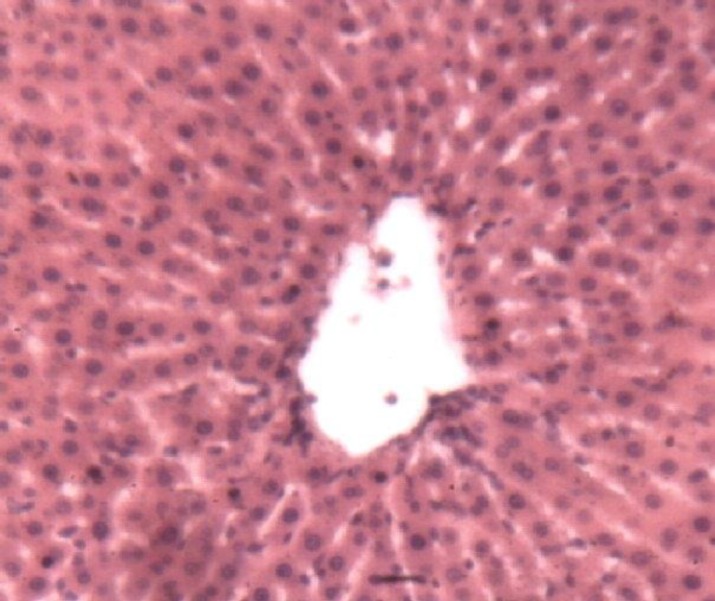
Histological liver of the rats treated with fruits at a dose of 400 mg/kg. Legend: Near-normal lobular structure. Image taken at 10x magnification

**Figure 6f:**
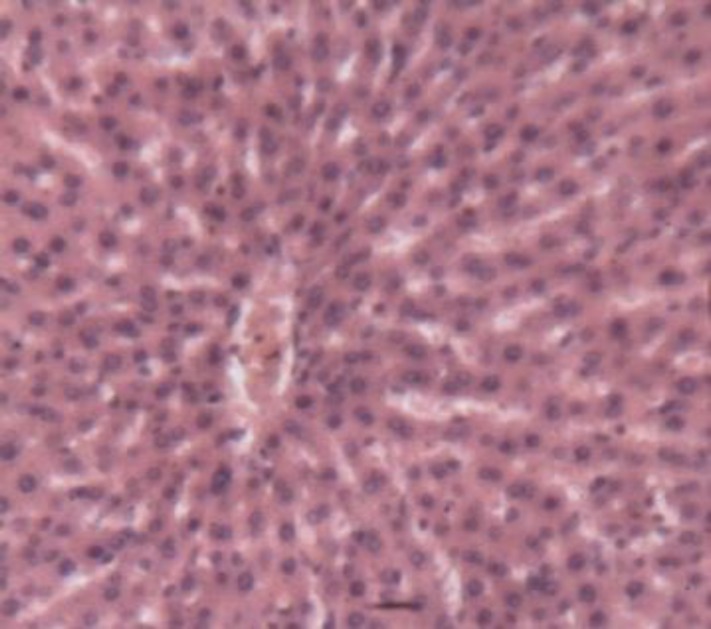
Histological liver of the rats treated with fruits at a dose of 800 mg/kg. ructure. Image taken at 10x magnification

**Figure 6g:**
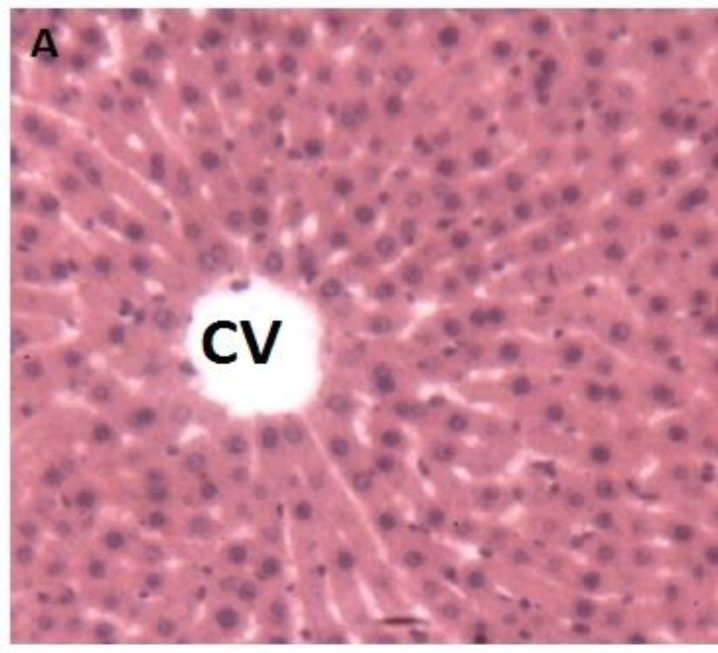
Histological appearance of the liver of rats treated with the reference drug (Atorvastatin).

Legend: Almost normal lobular structure. A Central Vein (CV) is shown in Figure A. Image taken at 10x magnification.

**Figure 6h:**
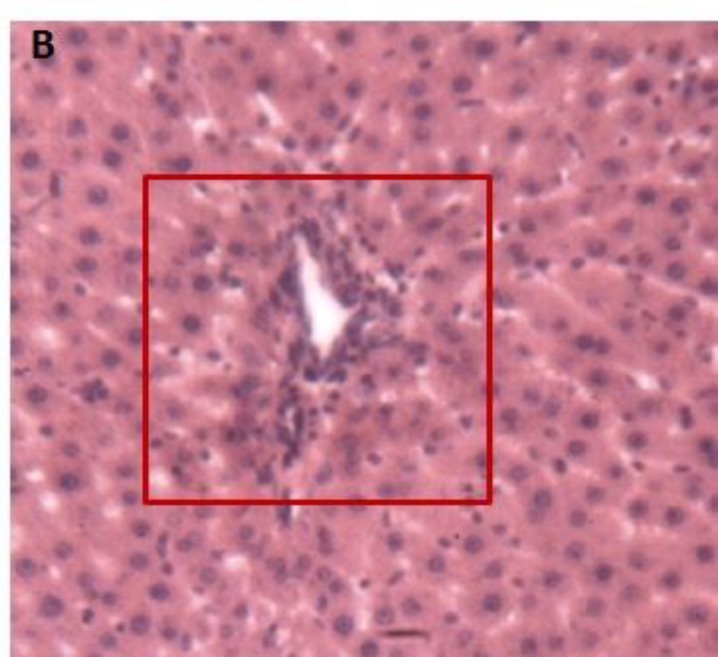
Histological appearance of the liver of rats treated with the reference drug (Atorvastatin).

Legend: Almost normal lobular structure. Space door is framed in Figure B. Image taken at 10x magnification.

## Discussion

Total cholesterol is the basic parameter of the lipid’s research. It is found in small proportion from foodborne and for the most part produced by the liver. Its assay can detect isolated hypercholesterolemia or hypercholesterolemia associated with hypertriglyceridemia (Odou, 2013 ▶). 

The peak observed in the positive control group showed that Triton X-100 has created the expected hypercholesterolemic disorder. This is the same conclusion which led several authors who performed the same protocol with a single dose of 150 mg/kg (Kothiyal and Gupta, 2011 ▶) while others used a single dose of 100 mg/kg (Ghule et al, 2006 ▶; Keshetty et al., 2009 ▶; Sudha et al., 2011 ▶; Patel et al., 2012 ▶). Indeed, Triton X-100 is a nonionic detergent that causes elevation of total cholesterol and triglycerides in the blood altering hepatic lipid metabolism (Patel et al., 2012 ▶). Although a dose-related effect was not observed, leaves and fruits, whatever the dose used, significantly reduced the total cholesterol. 

The traditional use of the fruits of this vegetable in Nigeria against hypercholesterolemia (Sodipo et al., 2012 ▶) is then justified. Beyond the work of Sodipo et al. (2012) ▶ on Nigerian fruits of *S. macrocarpon*, this work proved that both the leaves and fruits of this vegetable produced in Benin have a cholesterol lowering effect. As the group treated with the reference drug having experienced the same effect, it can be assumed that the powder of *S. macrocarpon* as cholesterol-lowering agent, would be preferentially selective and competitive inhibitor of HMG-CoA reductase, which is responsible for the synthesis of cholesterol by the liver enzyme. This results in the reduction of cholesterol and triglycerides and thus a reduction in cardiovascular risk. Regular consumption of leaves especially (due to the low presence of some toxic compounds such as solasodine found in fruits and which is supposed to disappear at high cooking temperature) is recommended as this vegetable contains high levels of proteins (Dougnon et al., 2012 ▶). 

This results suggests good prospects for treatment by a pharmafood as many plants used for the treatment of hypercholesterolemia [*Ruellia tuberosa* Linn (Krishna et al., 2012 ▶), *Medohar vati* (Patel et al., 2012 ▶)]. This cholesterol-reducing activity of *S. macrocarpon* is conferred by the presence of chemical compounds such as tannins found in the leaves as well as in fruits by promoting the production of bile and thus lipid digestion (Krishna et al., 2012 ▶; Dougnon et al., 2012 ▶). The saponins present in the leaves (Dougnon et al., 2012 ▶) also act as cholesterol-lowering agents by binding with cholesterol in the intestinal lumen (Ghule et al., 2006 ▶) which lowers circulating cholesterol. 

HDL-cholesterol is the fraction of cholesterol contained in HDL lipoproteins. It is recognized that it is the "protective" cholesterol fraction because there is an inverse relationship between the concentration of HDL-cholesterol and the incidence of cardiovascular complications. HDL lipoprotein is involved in the regulation of cholesterol (Odou, 2013 ▶). The rats in the control group experienced significant decrease in HDL-cholesterol following induction of hypercholesterolemic disorders by Triton X-100. The group treated with the drug and those treated with the powder of leaves and fruits showed a high level of HDL cholesterol, which shows that consumption of *S. macrocarpon* is associated with a decrease in the risk of atherosclerosis. In fact, an elevation of HDL-cholesterol is associated with a decreased risk of atherosclerosis (Sodipo et al., 2011 ▶). A better effect was shown by the lot treated with powdered fruits at 800 mg/kg. This dose worked better than those of 400 mg/kg. 

This effect is due to an increase in the activity of lecithin that plays a key role in the incorporation of free cholesterol in HDL and VLDL which turn them back to the liver cells (Kothiyal and Gupta, 2011 ▶). It should be noted that HDL facilitates the transport of cholesterol from peripheral tissues such as arteries to the liver for catabolism (Venkatesham et al., 2009 ▶). The powders of leaves and fruits of *S. macrocarpon* caused a decrease in LDL-cholesterol. 

The consumption of this vegetable would then reduce the incidence of cardiovascular or coronary heart diseases (Kothiyal and Gupta, 2011 ▶). The powders of leaves and fruits of *S. macrocarpon* induce reduction of triglycerides and VLDL, which reinforces the assertion that this vegetable should be promoted and consumed to naturally fight against lipid disorders. The dose of powdered fruits at 400 mg/kg seems to be ineffective as those of 800 mg/kg or powdered leaves at 400 and 800 mg/kg. All these biochemical results are reinforced by histological study of the liver. Observed tissue repair is due to the process described above and this confirms that the powder of leaves and fruits of *S. macrocarpon* are hypocholesterolemic. 

*S. macrocarpon*, a vegetable widely producted at Cotonou has been evaluated for its cholesterol-lowering properties. The leaves and fruits were powdered and used to binge hypercholesterolemic rats created by peritoneal injection of Triton X-100. The results suggested the protective role of this plant against cardiovascular diseases which significantly lowers the total cholesterol, LDL-cholesterol, VLDL-cholesterol, and triglyceride levels while significantly increases HDL cholesterol. This shows how the African flora is rich in natural substances with a therapeutic range. A diet rich in vegetables such as *S. macrocarpon* should be recommended.
